# Neonatal disease environment limits the efficacy of retinal transplantation in the LCA8 mouse model

**DOI:** 10.1186/s12886-016-0368-0

**Published:** 2016-11-04

**Authors:** Seo-Hee Cho, Ji Yun Song, Jinyeon Shin, Seonhee Kim

**Affiliations:** Department of Anatomy and Cell Biology, Shriners Hospitals Pediatric Research Center, Lewis Katz School of Medicine at Temple University, 3500 N. Broad Street, Philadelphia, PA 19140 USA

**Keywords:** LCA8, Cell therapy, Retina, Rosette, Host response

## Abstract

**Background:**

Mutations of Crb1 gene cause irreversible and incurable visual impairment in humans. This study aims to use an LCA8-like mouse model to identify host-mediated responses that might interfere with survival, retinal integration and differentiation of grafted cells during neonatal cell therapy.

**Methods:**

Mixed retinal donor cells (1 ~ 2 × 10^4^) isolated from neural retinas of neonatal eGFP transgenic mice were injected into the subretinal space of LCA8-like model neonatal mice. Markers of specific cell types were used to analyze microglial attraction, CSPG induction and retinal cell differentiation. The positions of host retinal cells were traced according to their laminar location during disease progression to look for host cell rearrangements that might inhibit retinal integration of the transplanted cells.

**Results:**

Transplanted retinal cells showed poor survival and attracted microglial cells, but CSPG was not greatly induced. Retinas of the LCA8 model hosts underwent significant cellular rearrangement, including rosette formation and apical displacement of inner retinal cells.

**Conclusions:**

Local disease environment, particularly host immune responses to injected cells and formation of a physical barrier caused by apical migration of host retinal cells upon disruption of outer limiting membrane, may impose two major barriers in LCAs cell transplantation therapy.

## Background

Photoreceptor degeneration leads to irreversible blindness in related retinal degenerative diseases, including Retinitis Pigmentosa (RP) and Leber Congenital Amaurosis (LCA) [1, 2]. The early-onset retinal degeneration (RD) responsible for LCA, the severe disease leading to childhood blindness in from 1 in 30,000 to 1 in 81,000 subjects, is caused by mutation in approximately 22 genes implicated in photoreceptor function, structure and development [1, 3, 4]. Although no effective treatment or cure is currently available, recent advances in cell transplantation have greatly improved the survival, integration and differentiation of donor cells as well as functional recovery in animal models [5].

Two major barriers to cell-based ocular therapy are intrinsic limitations of donor cells that affect survival, migration and differentiation, and adverse host-mediated reactions to subretinal injection [6–8]. Host-specific limitations include the physical barrier to transplanted cell migration imposed by the outer limiting membrane (OLM, also known as an external limiting membrane) located at the apical side of the outer nuclear layer (ONL). Although OLM is formed at the junctions between rod photoreceptors and Muller glial cells by junction proteins and junction - associated proteins, including Crumbs polarity complex proteins [9–11], the precursor of OLM is detected in early retina before these cells have been generated. Therefore, it is plausible that junctions between retinal progenitors are essential to maintain embryonic and neonatal retinal integrity during development. The local immune reaction also affects the survival of transplanted cells. In developing and mature retina, microglia (MG) control immune surveillance and homeostasis [12]. Pathogenic activation of MG is correlated with the progression of RD [13–15] and pharmacological immunosuppression, including of MG activation, is protective in the RD model [16] and enhances the survival of donor cells [17]. In general, host responses to degenerating retinal cells are highly variable and disease-specific [6, 18–20].

Human LCA type 8 (LCA8) is caused by Crb1 gene mutation [21, 22]. The most striking phenotype among related LCA8 diseases is early-onset retinal laminar disorganization triggered by the loss of cell to cell adhesion, presumably between progenitor cells, at the apical surface of the embryonic retina. In late-onset RP12 similar Crb1 genetic defects are thought to disrupt photoreceptor to Muller glial adhesion at the apical surface of the mature retina. A partial break in OLM, a major barrier to cell migration [9, 23], appears to enable efficient integration of transplanted cells into host retina in Crb1^rd8/rd8^ retinas [6]. Partial chemical breakdown of OLM has also been reported to facilitate retinal integration [8]. However, LCA8-like mouse models with embryonic onset, such as Crumbs 1 and/or 2 (Crb1 and/or 2) mutant mice and conditional knock out (CKO) of protein associated with lin seven-1 (Pals1, also known as MPP5), an interacting protein with Crumbs homologs, exhibit robust cellular rearrangement during and after embryonic development following disruption of cell adhesion [24–27]. In Pals1 CKO retinas, apical localization of Crumb homologs, Par3 and β-catenin is severely disturbed during and after retinal development, and embryonic retinal cells and photoreceptor cells rearrange into half-rosettes or rosettes. Retinal cells, especially inner nuclear layer (INL) and ganglion cell layer (GCL) cells, are also apically displaced. These apically displaced cells include ganglion and amacrine cells, and Muller glia cell bodies, which are normally located centrally. All of these displacements suggest that apico-basal tissue polarity is lost due to impaired cell-cell attachment. The extensive structural defects cause ERG responses of Pals1-deficient eyes to be severely reduced or virtually absent. Laminar disorganization during Pals1 CKO pathogenesis is likely to provide an adverse environment for cell-based therapy, but this notion has not been evaluated.

This study uses Pals1 CKO as a model to identify components of the neonatal retinal environment that adversely affect cell transplantation in degenerating retinas undergoing laminar disorganization. The investigation identified two major host responses that predominantly inhibited the survival and integration of transplanted cells: the MG-mediated immune response and retinal rearrangement that opposed migration into the retina.

## Methods

### Animals and preparation of retinal cells

All animal procedures discussed in this manuscript for the ethical treatment of animals including handling, housing, surgeries, post-surgical monitoring, anesthesia and euthanasia were approved by the Institutional Animal Care and Use Committee at Temple University. eGFP (+) mice (C57BL/6-Tg(CAG-EGFP)10sb/J, The Jackson Laboratory) were genotyped under a fluorescent microscope. Pals1^f/f^ allele and Rx-Cre line were previously described [26]. PCR-based genotyping was performed using the primers as described. Pals1 CKO (PR; Pals1 ^f/f^; Rx-Cre), heterozygote (Pals1^f/+^; Rx-Cre) and wild type (WT) control littermates (mice not carrying Rx-Cre) were typically obtained from the crosses of heterozygotes and used for the assays. Swiss Webster (SW) mice potentially containing rd^1^ allele (The Jackson Laboratory) were used as additional hosts for cell transplantation experiments.

### Subretinal injection via transcleral route

eGFP (+) retinas were dissected from P0-P5 eGFP (C57BL/6-Tg(ACTB-EGFP)1Osb/J) strain (The Jackson Laboratory). Whole retinal cells were dissociated after 5 min trypsin incubation at 37 ^o^C followed by trituration, and diluted to a final concentration of ~2 × 10^4^ cells/μl. One μl cell suspension containing approximately 1 ~ 2 × 10^4^ cells and fast green dye (0.1 %) was injected into the subretinal space of the host at neonatal stages between P2 and P6 using a capillary needle via the transcleral route [28]. Retinas were mainly analyzed between P21 (*n* > 3) and P35 (*n* > 5). For SW, retinas were analyzed at P21 (*n* = 5). Due to substantial variation in the size of eGFP (+) grafts, which probably resulted from methodological difficulties associated with neonatal subretinal injection, and technical challenges determining total cell numbers in grafts, this study relied on qualitative, not quantitative, assessments of grafted cells and host responses.

### Confocal imaging of flat-mount retinas, immunofluorescence assays of sections and 3D reconstruction of stacked images

Eyes showing green fluorescence were prescreened under a fluorescent microscope after enucleation. For flat-mount analysis, eGFP (+) retinas were fixed in 4 % paraformaldehyde for 2 h. After removing overlying retinal pigment epithelium (RPE), choroid and sclera, retinas were dissected out from the lens. Five corners of the retinas were cut with a spring scissors to flatten the retina, which was subjected to permeabilization (0.5 % dimethyl sulfoxide (DMSO), 5 % Triton in phosphate buffered saline) and blocking (3 % goat serum, 0.5 % DMSO, 5 % Triton in tris-buffered saline). Primary antibodies in blocking buffer were incubated with retinas for 3 days, and then incubated with secondary antibody overnight. Retinas stained with Hoechst 33258 were mounted with Fluoromount-G (SouthernBiotech.) on the slide (with photoreceptor side up) for confocal imaging (TCS SP8, Leica Microsystems GmbH, Germany). Stacks of images covering approximately half of the retinal thickness (2 μm steps) were typically taken from the photoreceptor side. Imaris (Bitplane) was used for 3D reconstruction of the stacks of confocal images. For section antibody staining, eGFP (+) eyeballs were fixed in 4 % paraformaldehyde and dehydrated through an ethanol series. Seven-micron thick paraffin sections were cut, and transplanted cells were detected by anti-GFP staining.

### Antibodies

Primary antibodies: anti-Ceh-10 homeodomain-containing homolog (Chx10, also known as VSX2) (Exalpha, X1179p), anti-CS56 (Abcam, ab11570), anti-GFP (Life, G10362 (rabbit); Aves labs, GFP-1020 (chick)), anti-GS (BD, 610517), anti-Iba I (Wako, 019-19741), anti-Pax6 (Abcam, ab5790), anti- phosphosynaptic density protein 95 (PSD-95) (CST, #3409), anti-rhodopsin (Phosphosolutions, 1840-RHO), and anti-Sox9 (Millipore, ab5535). Secondary antibodies: Alexa488 conjugated donkey anti-rabbit (Jackson Immuno Res., 711-545-152) and Alexa488 conjugated goat anti-mouse (Life, A-11029), Alexa488 conjugated goat anti-chick (Invitrogen, A11039), Cy3 goat anti-rabbit (Jackson Immuno Res., 111-165-047 or 111-165-144), Alexa647 conjugated goat anti-mouse (Life, A21236) and Alexa647 conjugated donkey anti-rabbit (Life, A31573) antibodies.

## Results

### Transplantation of eGFP (+) retinal cells into neonatal Pals CKO retinas

**Fig. 1:**
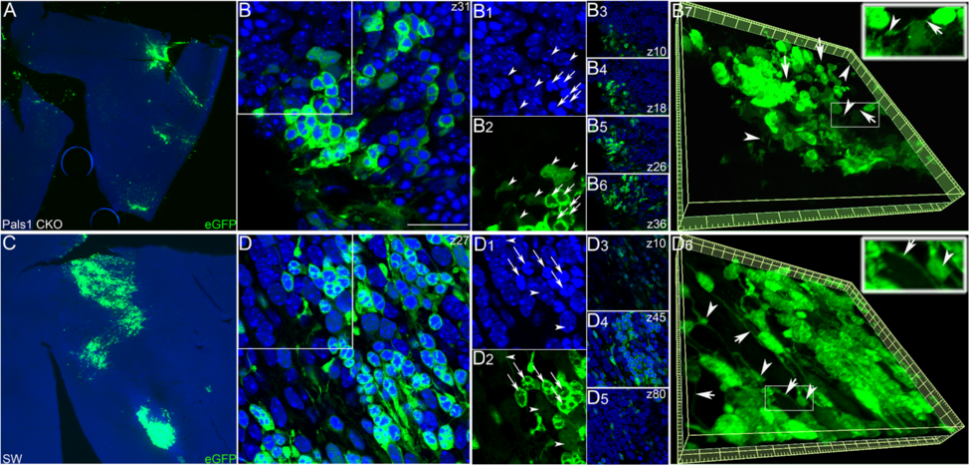
(A) Representative flat-mount image showing eGFP (+) cell clusters (*green*) on the photoreceptor side of a Pals1 CKO at P24. (B – B2) Confocal image at z31 (z stack number) reveals two different Hoechst dye and GFP staining patterns for rods (*arrows*) and INL cells (*arrowheads*). (B3 – B6) Analysis of confocal image stacks separated by 4, 4 and 5 um (z (stack number) = 10, 18, 26 and 36, with 0.5um interval) shows multiple cell layers in GFP (+) clumps. (B7) 3D reconstruction of confocal images used in B – B6 shows cellular processes (*arrows*) and synaptic termini (*arrowheads*). See magnified inset for detail. (C) Representative flat-mount image showing eGFP (+) cell clusters (*green*) on the photoreceptor side of a SW at P21. (D – D2) Confocal image reveals two different Hoechst dye and GFP staining patterns for rods (*arrows*) and INL cells (*arrowheads*). (D3 – D5) Confocal image stacks separated by 3.5 and 3.5 um (z (stack number) = 10, 45 and 80, with 0.1um interval) show multiple cell layers in GFP (+) clumps. (D6) 3D reconstruction of confocal images used in D – D5 shows cellular processes (*arrows*) and synaptic termini (*arrowheads*). See magnified inset for detail. Hoechst dye signal in D1 was amplified to visualize INL cells, which usually show low intensity. Scale bars, 25 μm

LCA8 is characterized by early-onset visual impairment associated with disruption of OLM and subsequent retinal folding and rosette formation. In order to evaluate survival of transplanted cells in the LCA8 disease environment, approximately 20,000 dissociated enhanced green fluorescent protein (eGFP) (+) retinal cells from neonatal C57BL/6-Tg(CAG-EGFP)10sb/J) pups (P0 – P5) were subretinally injected into neonatal Pals1 CKO retinas. This model mimics the critical pathophysiology of human LCA8 caused by Crb1 mutations [26]. Host retinas were first examined at P21 or P35 in retinal flat-mount preparations using stacked images obtained with a confocal microscope. In general, the areas occupied by engrafted eGFP (+) cells varied greatly (Fig. 1A; P21 (*n* > 3) and P35 (*n* > 5)), and transplanted cells displayed two different levels of eGFP fluorescence intensity (Fig. 1B). The most prevalent eGFP (+) cell type had a characteristic Hoechst 33258 staining pattern resembling that of rods [29, 30] and a strong eGFP signal. These cells also showed two to three brightly stained chromocenters, strong cytoplasmic eGFP signal and bipolar processes with termini (Fig. 1B1). The other cell type was typically larger than rods and demonstrated a weak eGFP signal and several small chromocenters, which is a characteristic of INL cells (Fig. 1B1). Confocal analysis and 3D reconstruction of images from Pals1 CKO showed that transplanted eGFP (+) cells formed multi-layered cell clumps (from a single cell to 3-5 layered cells in thickness), and cellular processes in host retinas similar in structure to synaptic termini (Fig. 1 B3 – B7). In parallel, to compare the survival of eGFP (+) transplanted cells in control animals, retinas of SW pups received grafts that were injected, harvested and examined in a similar fashion. As in Pals1 CKO, eGFP (+) cell clumps showed two nuclear Hoechst 33258 staining patterns and two eGFP staining patterns (Fig. 1C - D2). In addition, eGFP (+) transplanted cells formed multi-layered clumps, and extensive bipolar processes and synaptic termini, which resembled the features of photoreceptor cells (Fig. 1D3 – D6). In summary, multi-layered grafted eGFP+ retinal cells similarly formed two distinctive patterns following transplantation into either SW or Pals1 CKO hosts.

### Retinal integration

**Fig. 2:**
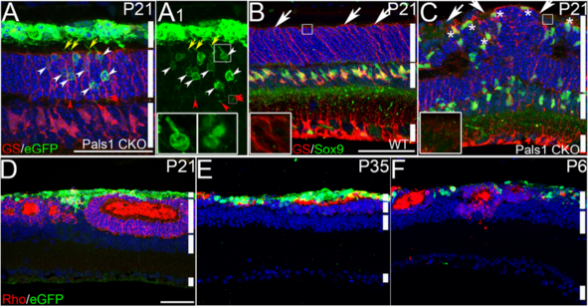
Impaired retinal integration of eGFP (+) donor cells in Pals1 CKO retinas. (A & A1) An example of a cell transplant in a Pals1 CKO P21 retinal region without evident ONL disorganization. eGFP (+) cells migrate into ONL (*white arrowheads*) and form synaptic termini (*red arrowheads*) and bipolar processes (*yellow arrows*). *White bars* represent subretinal space/inner and outer segments, ONL and INL (*from top to bottom*). Insets in A1 show photoreceptor cell body and synaptic termini. (B & C) Representative images showing Muller glial disorganization, including apical surface (*arrows*) and misplaced cell body (*), in Pals1 CKO retina (C) compared to WT (B). Muller glial cells are marked with α-GS (*red*) and α − Sox9 (*green*). *White bars* represent ONL, INL and GCL (*from top to bottom*). Insets show apical endfeet of Muller glial cells. (D) An example of eGFP (+) cells transplanted in a rosette forming retinal region showing poor retinal integration at P21. (E & F) Examples of eGFP (+) donor cells transplanted into Pals1 CKO retinas harvested at P35 (E) and P60 (F) do not exhibit increased retinal integration. To help retinal orientation, Muller glial cells (A), rod cells and segments (D – F) are stained with α-GS and α-rhodopsin antibodies, respectively (*red*). White bars represent subretinal space/inner and outer segments, ONL, INL and GCL except F, where subretinal space/inner and outer segments overlap with outer segment (*from top to bottom*). Hoechst 33258 staining (*blue*) is shown for nuclear counterstaining. Scale bars, 50 μm

We next studied the profile of retinal migration of the donor cells in Pals1 CKO. In order to assess the progressive changes, paraffin sections of retinas were analyzed at P21, P35 and P60. Examination of donor cells in P21 Pals1 CKO retinas, when primary retinal development is completed, revealed two different phenotypes. In the first phenotype, a subset of eGFP (+) transplanted cells had migrated in the ONL (Fig. 2A - A1), but most remained in the subretinal space. This phenotype occurred in areas of Pals1 CKO retina that appeared normal, where Muller glial arrangement was unaltered compared to WT, and which clearly differed from areas of Pals1 CKO retina that were severely affected (Fig. 2A – C). For example, the location and distribution of Muller cell bodies (Sox9 (+)) in this unaffected Pals1 CKO retinal region resembled WT more closely than they resembled the severely affected Pals1 CKO region, where Muller glial cell bodies were apically displaced and the apical surface, including OLM, was severely disorganized [26]. Mosaic expression of Cre in Rx-Cre driver most likely accounted for the phenotypic variation in Pals1 CKO [26]. Migrated cells formed structures reminiscent of rod cells, including bipolar processes and rod termini, at the bottom of ONL.

In the other predominant phenotype, a majority of donor cells failed to migrate into the host retina (Fig. 2D). Instead, they formed clumps between retinal rosettes and RPE or in or around superficial retinal areas separated by rosettes (Fig. 2D1-D2). The latter phenotype preferentially appeared around retinas undergoing active rearrangement exemplified by folding or rosette formation at P21. The predominantly subretinal retention of donor cells was largely unaltered at later stages (at P35 (*n* > 5) and P60 (*n* = 3)) (Fig. 2E & F).

### Differentiation of transplanted cells

**Fig. 3:**
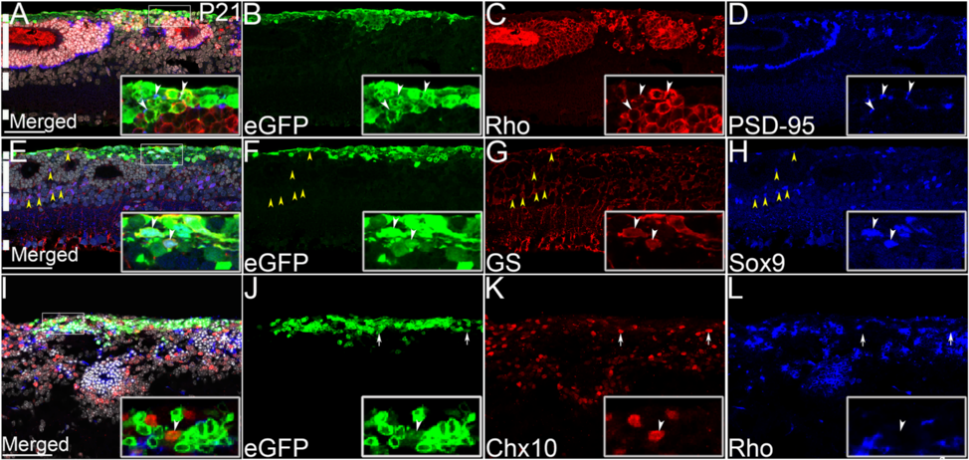
Section-antibody staining shows that transplanted eGFP (+) donor cells (*green*) differentiate into retinal cells in Pals1 CKO at P21. **a** – **d** Subsets of eGFP (+) cells (*arrowheads in the box*) express rhodopsin (*red*), and post-synaptic marker, PSD-95 (*blue*). **e** – **h** Subsets of donor cells (*white arrowheads*) differentiate into cells that express GS (*red*) and Sox 9 (*blue*), Muller glia markers. Examples of endogenous Muller cells are marked with yellow arrowheads. **i** – **l** Subsets of donors (*arrows*) express Chx10, progenitor/ bipolar cell marker (*red*). Images used in the insets are from one confocal scan while large images are maximally projected. White lines indicate subretinal space/inner and outer segments, ONL, INL and GCL (**a & e**, *from top to bottom*). Dysmorphic retinal morphology precludes definite identification of retinal layers in I. Hoechst 33258 staining (*gray*) is shown for nuclear counterstaining. Scale bars, 50 μm

We next examined the extent of retinal differentiation by immunofluorescent antibody staining of paraffin sections. Most eGFP (+) cells that migrated into Pasl1 CKO retinas were morphologically similar to rods: bipolar processes emanated from the cell body, and the location and shape of the termini were consistent (Fig. 2A - A1). We also examined clumped cells that failed to migrate into the retina with retinal cell-type specific markers. Most of the donor cells expressed the rod-specific gene, rhodopsin (Fig. 3a – c), and a subset of eGFP (+) rod cells showed the postsynaptic marker, PSD-95, in close proximity, suggesting maturation into rod photoreceptor cells (Fig. 3d). A subset of cells that remained in the subretinal space expressed glutamine synthetase (GS), a marker of Muller glia (Fig. 3e – h). However, transplanted eGFP (+) Muller cells did not extend apical and basal processes encompassing the entire retina. Similarly, a small number of clumped cells expressed Chx10 (bipolar cells) (Fig. 3i – l) or Pax6 (ganglion, amacrine and horizontal cells; result not shown). eGFP (+)/Chx10 (+) or eGFP (+)/Pax6 (+) cells were not fully integrated into the recipient retina and were mainly localized in the subretinal space.

### Host-mediated responses to cell transplantation

**Fig. 4:**
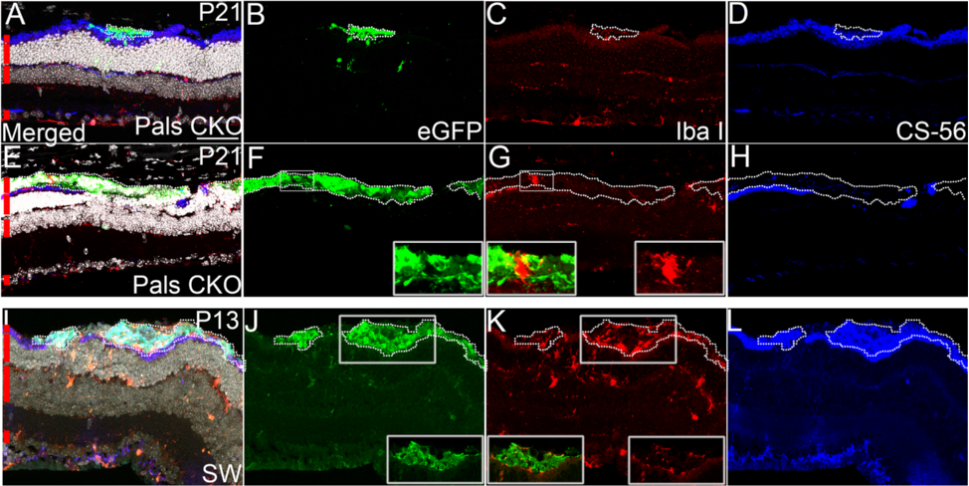
Transplanted eGFP (+) donor cells elicit mild host responses. **a** – **d** In normal retinal region of Pals1 CKO at P21, eGFP (+) cells recruit a small number of Iba I (+) cells (*red*, MG processes) and do not induce expression of CSPG (*blue*). **e** - **h** In degenerating retinal regions of Pals1 CKO at P21, small numbers of Iba I (+) MG cells (*red*) are recruited around transplanted cells while CSPG is not highly upregulated (*blue*, see below for comparison). Insets in **f** and **g** show high-mag views of an MG cell within the graft. **i** – **l** eGFP (+) donor cells recruit MG cells (*red*) and dramatically induce CSPG (*blue*) in host SW. Insets in **j** and **k** show MG cells located near transplanted eGFP(+) cells. *Red lines* indicate subretinal space/inner and outer segments, ONL, INL and GCL (*from top to bottom*). Hoechst 33258 staining (*gray*) is shown for nuclear counterstaining. Scale bars, 50 μm

eGFP (+) injected cells appeared to differentiate relatively normally within the subretinal space of the host (Fig. 3). This may be due to the intrinsic properties of the donor cells, which were enriched with late-stage progenitors and rod precursors, and to the inner-retinal environment of the neonatal host, which favors neurogenesis and neural differentiation. Some eGFP (+) injected cells integrated into the ONL of the recipient retina, but most remained in the subretinal space. Although a previous study showed that retinal integration largely occurs within 4 days post-transplantation in adult host mice [20], this may be caused by insufficient time for retinal migration after neonatal injection in neonatal LCA8-like model. When multiple transplanted clusters were examined at P35 (*n* > 5) and P60 (*n* = 3), retinal integration was not altered (Fig. 2). The non-permissive host environment provides another explanation. For example, host immune responses and the disease-specific host environment, alone or in combination, may significantly affect the fates of the injected cells. In order to assess these influences, two different retinal regions of Pals1 CKO, one normal looking and the other actively degenerating, were stained for evidence of MG activation and chondroitin sulfate proteoglycan (CSPG) induction at P21. To investigate MG activation, which plays a significant role in inhibiting effective cell transplantation [31], retinal sections were stained with α-Iba I antibody, which detects both resting and pathologically activated M1 MG [32–34]. In normal looking regions of the Pals1 CKO retinas, a small number of MG cells had been recruited by transplanted cells (Fig. 4a - c). In degenerating regions of Pals1 CKO retinas, Iba I (+) MG cells were intermixed with the injected cells (Fig. 4e – g, inset). The MG cells ectopically recruited in the subretinal space were hypertrophic, suggesting possible activation into the M1 stage. Therefore, it is likely that injected donor cells induced the recruitment, and possibly the activation, of MG cells in host retina. In parallel, normal SW retinas also attracted MG cells in and around grafted cells (Fig. 4i – k, insets).

We also examined the involvement of CSPG, a potent inhibitor of cell survival and migration that is normally expressed in the interphotoreceptor matrix [31, 35, 36]. When chondroitinase ABC was administered into the subretinal space of Rho^-/-^ mice simultaneously with a transplant, retinal migration was facilitated by breakdown of the glial scar and CSPGs [37]. Analysis of α-CS-56 immunoreactivity (specific to chondroitin sulfate types A and C, but not B) revealed that the CSPG level was lower in the host area filled with injected eGFP (+) cells than the level of endogenous CSPG in the interphotoreceptor matrix of the transplanted and non-transplanted neighboring areas (Fig. 4a, d, e and h). In order to assess whether this pattern of CSPG induction is a general feature of host retinas, we also examined SW retinas. SW retinas, when examined at P13 (*n* = 2) after neonatal transplantation of eGFP (+) cells, showed more robust CSPG induction than Pals1 CKO retinas, suggesting host-specific differences (Fig. 4i – l). In summary, the pathological environment in Pals1 CKO and SW retinas elicits MG recruitment upon cell transplantation, but Pals1 CKO retinas do not induce vigorous CSPG expression.

### Cellular rearrangement in Pals1 CKO during and after retinal development

**Fig. 5:**
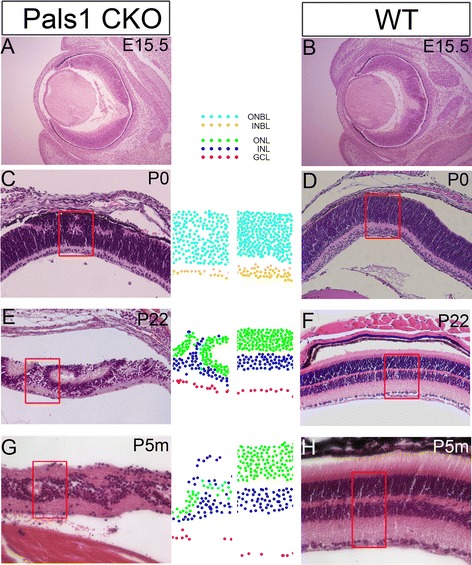
Progressive cellular rearrangement of host retina. **a** – **h** Representative H&E images illustrate abnormal localization of the retinal cells during disease progression according to the retinal layer information. **a**, **c**, **e** and **g** Retinal cells located inside the *red rectangles* are traced using different color codes (see legend) based on their laminar locations (INBL vs. ONBL at P0; ONL, INL and GCL at P22 and P5 months old) during disease progression from E15.5 to 5 month-old adult. **b**, **d**, **f** and **h** Similarly analyzed WT retinas at corresponding stages are used for comparison

Although induction of the MG-mediated immune response may affect survival of the transplanted cells, other inhibitory mechanisms may also play significant roles in causing integration to be much less efficient in degenerating regions of Pals CKO retina than in WT or non-affected regions. One potential mechanism is the physical forces formed during retinal resetting. For example, host (resident) retinal cells in degenerating retinas might exert resisting force by moving apically, which would cause transplanted cells migrating basally for retinal integration to encounter physical traction. A previous study showing apically displaced INL cells supports this hypothesis [26]. In order to test this possibility, we traced the position, from embryonic to adult, of host retinal cells during degeneration and concomitant retinal rearrangement according to their laminar locations in ONL, INL and GCL. We used positional information, size and H&E staining intensity to identify residential retinal layers. For example, rod cells are the smallest and show dark purple staining, while INL cells are intermediate in size and staining. GCL cells are in general the largest and located at the bottom of the epithelium. As shown in Fig. 5, Pals1 CKO retinas at E15.5 and P0 did not show any severely ectopic host cells in the outer or inner neuroblastic layers (ONBL and INBL) despite the apparent rosette forming activity at P0 (Fig. 5a – d). At P22, many INL cells located among retinal rosettes were positioned apically compared to WT controls, and a few were located at the apical surface (Fig. 5e & f). Five-month old Pals1 CKO retinas showed GCL cells and, to a lesser degree, INL cells, abnormally localized at the apical surface (Fig. 5g & h). These observations are consistent with our previous report showing partial duplication of the retina in postnatal retinas of Pals1 CKO [26].

In summary, host retinal properties of Pals1 CKO may impose two major inhibitory barriers to transplanted cells. First, potentially pathological MG cells are recruited to the injected site. In addition, retinal cellular arrangement during rosette formation may oppose a strong inhibitory force to the retinal integration of transplanted cells. Because subretinal cell injection induces CSPG in SW, but not in Pals1 CKO, intrinsic properties of the host retina and responses to the transplanted cells may together pose major obstacles to retinal cell transplantation in LCA8 models.

## Discussion

LCA8 is unique among the approximately 20 subtypes of LCA in that it is caused by mutations in apical polarity complex gene, Crb1 [1, 2, 24, 37]. As a result, affected retinas show destabilized OLM, pseudorosettes and thickening of the central retina (parafovea). Intriguingly, most of the human phenotype is recapped in mouse mutants not only of Crb1 gene, but also of Crb2, homolog and Pals1, interacting protein [24–26]. It is also interesting that human Crb1 mutations located at extracellular and intracellular domains induce milder late-onset RP12 or severe early-onset LCA8 without an obvious genotype-phenotype correlation [21]. Although the onset and severity of these two diseases are significantly different, both are caused by defects in retinal structural integrity. In rd^8^/rd^8^, a spontaneous frame-shift mutant of Crb1 and a mouse model for RP12, retinal lesions are focal and caused by failure to form cell-to-cell attachment between rod photoreceptor cells and Muller glia [9, 11]. In other mouse models partially mimicking human LCA8 pathology, abnormalities are observed in early embryonic retinas. Because the genesis of the majority of the rods and Muller glia starts postnatally [38, 39], retinal laminar disorganization is likely caused by attachment failure between progenitor cells. Also, in contrast to RP12, in LCA8 the initial cellular detachment occurs in developing retina while cells are born and migrate via interkinetic nuclear migration, and while the retina is growing horizontally. The extensive horizontal growth of the retina can magnify the effects of loss of cellular attachments.

Examination of whole-mount sections in the present study shows that eGFP (+) retinal cells, which contain late-stage progenitors, precursors of rods and Muller glia and late-born amacrine cells in addition to postmitotic retinal neurons, form clumps whose area varies enormously in Pals1 CKO and SW retinas. The size of the clumps is presumably affected by subretinal targeting efficiency and survival of the transplanted cells. Therefore, we analyzed the fates of the transplanted cells and host responses qualitatively rather than quantitatively. We found that host retinal organization greatly influenced retinal integration of transplanted cells; unaffected or partially affected Pals1 CKO retinas showed facilitated migration of eGFP (+) cells, whereas migration was severely inhibited in retinal areas dominated by rosettes and/or laminar disorganization. Cells in the clumps expressed characteristic retinal markers, such as rhodopsin (rods), Pax6 (amacrine, horizontal and ganglion cells), Chx10 (bipolar cells) and GS (Muller glia). This expression pattern can be interpreted as evidence that transplanted cells clumped in the subretinal space differentiated normally. This view is supported by our observation of the terminal rod marker, PSD-95, in subsets of the transplanted cells and of rod cell processes and synaptic termini in 3D reconstruction images of the grafts. However, because Pax6 and Chx10 are expressed in retinal progenitors and precursors of retinal interneurons and bipolar cells, this result may simply suggest that donor cells within the graft maintain retinal gene expression. Despite the terminal differentiation, the morphology of the grafted clumped cells in general was severely defective. GS (+) Muller glial cells in the clumps, for example, did not demonstrate bipolar processes or expanded endfeet. Nevertheless, these injected cells were contained in the subretinal space of the host retinas throughout the stages examined, up to P60, more than 55 days after injection. Therefore, it is highly likely that the host-specific disease environment affects the migration of the injected cells. Factors suppressing the effectiveness of the cell transplantation therapy include the host immune response and activation of cell death, in addition to intrinsic properties of the donor cells.

One formidable host response is MG-activation, which not only inhibits donor cell integration into the retina, but also triggers apoptotic processes by a pro-inflammatory mechanism [40, 41]. Diseased host retinas such as Pals1 CKO show the signs of MG activation as early neonates. Breakdown products of retinal cells at the apical surface may play a direct role in recruiting resting MGs. Heightened immune surveillance by the diseased host in response to cell transplantation may therefore supplement the inhibition due to donor cells, which can facilitate pathogenic activation of MG cells via cellular proteins, fragments and debris. In addition, inherent rosette forming activity in developing Pals1 CKO retinas plays an important role in suppressing retinal integration of the transplanted cells. Tracing of layer-specific cells in Pals1 CKO retinas during disease progression showed the preferred apical positioning of the resident cells in degenerating retinas, especially in the areas between rosettes. It is conceivable that ONL cells in Pals1 CKO, which are held together by OLM after random breakdown of cell attachments, form a rosette when the developing retina grows horizontally. To a lesser degree, INL and GCL cells, which are in the process of forming columnar circuits, also responded to rosette formation mediated by ONL by shifting their position apically. In extreme cases GCL cells were located at the apical, rather than the basal, side of the diseased retina. This interpretation is consistent with the observation of ectopic Pax6 and β-Tubulin III (+) cells in the apical surface of the retinas by immunofluorescence antibody staining [26].

**Fig. 6:**
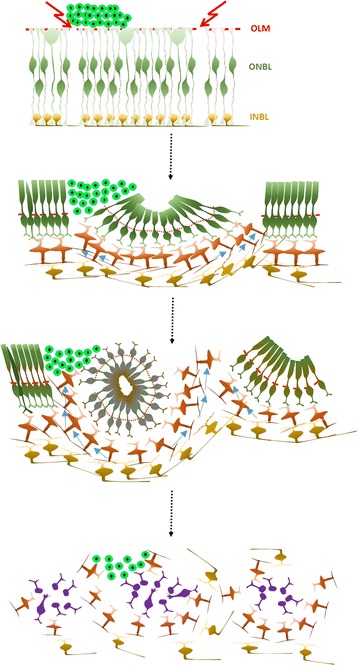
Schematic demonstrating retinal disorganization/reorganization in Pals1-deficient retinas (see text for detail). *Red arrows* denote the initial breaks in cell-to-cell attachment at the apical surface of embryonic or early neonatal retinas. *Blue arrows* indicate the direction of the cell movement that pushes INL and GCL cells toward the apical surface during retinal rosette formation. *Green* cells located in the subretinal space (top of the epithelium) denote eGFP (+) donor cells

During retinal development in LCA8, cell-to-cell adhesion is lost at the apical surface of the epithelium due to destabilization of Crumbs polarity complex [26]. Contact between retinal progenitor cells can be lost at an early stage, but contact between photoreceptor cells and Muller glia can also be lost at a late stage and after development is complete. In LCA8 and LCA8 animal models, the initial defects start during embryonic development (Fig. 6). When the loss of cellular attachment is extensive, ONL cells located between detachment points start to form a half-rosette due to horizontal retinal growth and apical constrictions among cells holding together. This process is further intensified to form a full rosette as development continues and results in delayed or abnormal photoreceptor maturation. The size of the rosettes varies and is determined by the distance between the two points at which extensive attachment is initially lost; there may be further division into smaller rosettes as more cellular detachment occurs within rosettes. During ONL rearrangement, INL cells and, to a lesser extent, GCL cells, are pushed apically through the gaps between rosettes [42], so that the retina not only loses laminar integrity but also fails to maintain proper circuitry. It is speculated that degeneration of retinal cells, including photoreceptors, is enhanced by abnormal oxidative stress. This stress is presumably imposed by excess oxygen due to decreased oxygen consumption and by improper nutrition due to secondary defects in blood supply. Retinal cellular rearrangement initiated by defects in cell-to-cell attachment may therefore impose an additional LCA8-specific inhibitory environment for cell transplantation therapy. Our results contrast dramatically with earlier reports demonstrating facilitated cell migration after retinal injection in animal models with partial breakdown of OLM [8]. These studies were performed in adult mice, in which mature retina may not exhibit significant rearrangement in response to localized cell-to-cell junctional breakdown, rather than in neonatal LCA8 retinas, in which robust developmental rearrangement follows retinal growth. The results with Pals1 CKO LCA8-like hosts also differed from those with SW hosts. In areas of SW retinas where rosettes were not formed, grafts both recruited MG and induced CSPG. However, in Pals1 CKO retinas, grafts attracted Iba1 (+) MG, but CSPG induction was minimal. This difference supports the notion that, in addition to undergoing the intrinsic cellular rearrangements that form retinal rosettes, the Pals1 CKO retina provides an environment with a distinctive host response to transplanted cells.

## Conclusions

This study identifies disease-specific factors that affect the survival and retinal integration of transplanted cells in the early-onset degenerative retinal disease LCA8. In addition to the general inhibitory host responses, such as MG activation, the neonatal LCA8 environment may impose a physical restraint due to cellular rearrangement in a degenerating retina with partly broken junctions essential for tissue integrity. Customized interventions designed to overcome these inhibitory host barriers will be essential for successful ocular cell-based therapy for LCA8.

## Abbreviations

CKO: Conditional knock-out
CSPG: Chondroitin sulfate proteoglycan
DMSO: Dimethyl sulfoxide
GCL: Ganglion cell layer
INL: Inner nuclear layer
LCA: Leber Congenital Amaurosis
MG: Microglia
OLM: Outer limiting membrane
ONL: Outer nuclear layer
RD: Retinal degeneration
RP: Retinitis pigmentosa
RPE: Retinal pigment epithelium
SW: Swiss Webster
